# An Intuitive Formulation of the Human Arm Active Endpoint Stiffness

**DOI:** 10.3390/s20185357

**Published:** 2020-09-18

**Authors:** Yuqiang Wu, Fei Zhao, Wansoo Kim, Arash Ajoudani

**Affiliations:** 1 State Key Laboratory for Manufacturing System Engineering, Xi’an Jiaotong University, Xi’an 710049, China; ztzhao@mail.xjtu.edu.cn; 2 Shaanxi Key Laboratory of Intelligent Robots and School of Mechanical Engineering, Xi’an Jiaotong University, Xi’an 710049, China; 3 Human Robot Interfaces and physical Interaction lab (HRI^2^), Istituto Italiano di Tecnologia (IIT), 16163 Genova, Italy; wan-soo.kim@iit.it (W.K.); arash.ajoudani@iit.it (A.A.)

**Keywords:** human factors, physical human–robot collaboration, robot adaptation and learning

## Abstract

In this work, we propose an intuitive and real-time model of the human arm active endpoint stiffness. In our model, the symmetric and positive-definite stiffness matrix is constructed through the eigendecomposition $K_{c}=VDV^{T}$, where V is an orthonormal matrix whose columns are the normalized eigenvectors of $K_{c}$, and D is a diagonal matrix whose entries are the eigenvalues of $K_{c}$. In this formulation, we propose to construct V and D directly by exploiting the geometric information from a reduced human arm skeleton structure in 3D and from the assumption that human arm muscles work synergistically when co-contracted. Through the perturbation experiments across multiple subjects under different arm configurations and muscle activation states, we identified the model parameters and examined the modeling accuracy. In comparison to our previous models for predicting human active arm endpoint stiffness, the new model offers significant advantages such as fast identification and personalization due to its principled simplicity. The proposed model is suitable for applications such as teleoperation, human–robot interaction and collaboration, and human ergonomic assessments, where a personalizable and real-time human kinodynamic model is a crucial requirement.

## 1. Introduction

The current generation of robotic manipulators has triggered new application scenarios in which robots can enter into direct interactions with unknown environments or humans to perform complex tasks [1]. The theory of impedance control [2] has played an important role in this direction, to control the dynamic interactions between a manipulator and the external world. In fact, due to its implementation simplicity and the robustness to the external disturbances, it has found several significant areas of application [3,4,5]. Nevertheless, the choice of impedance parameters in this control framework is not intuitive for a given task, especially for the tasks that require continuously varying interaction patterns [6,7,8].

To address the impedance planning problem from a machine learning point of view, previous works proposed the use of model-free reinforcement learning (RL) algorithms. In [6], a policy improvement with path integrals (PI^2^) algorithm was proposed to accomplish variable impedance control for a given task. In [8], the authors propose exploiting the end-effector space for RL to learn the reference position and impedance profile for contact-rich tasks. Although these works demonstrated the potential of the RL approach to fulfill the impedance requirements of a task, they demanded a large number of trials (hundreds in [6] and even more in [8]) to train the models.

An alternative approach to the impedance learning through RL is learning from demonstration (LfD). In [9], a LfD-based algorithm was proposed to collaboratively assemble a wooden table with a human subject and a robot. The desired trajectory was retrieved through a task-parametrized Gaussian mixture model (TPGMM). In addition, by taking into account the sensed force information, a weighted least-squares (WLS) method was employed to estimate the stiffness matrix, which was used in the task reproduction. In [10], the authors used a similar framework but adopted a sliding window technique to carry out local stiffness estimation instead of WLS. However, in both methods, a first estimation of the stiffness matrix does not comply with the symmetric and positive-definite (SPD) constraints. In [11], benefiting from estimations of both the mean and the variance from kernelized movement primitives (KMP) [12], the authors consider the variance of the positional demonstrations as the task-related uncertainty, whose inverse then is used as the stiffness parameters in the controller. Force sensing is not required in this approach, and the assumption that variability of position and stiffness are directly related may not always be reasonable [7].

It is well known that human arm endpoint stiffness can be adapted to different task requirements and instabilities through changing posture [13] and muscle contractions [14]. In fact, human interaction skills can be defined by the ability to exploit the two mechanisms to match the arm endpoint stiffness to the task requirements. A preliminary investigation of this concept was carried out by the authors in [15], where a virtual tennis system was developed to estimate the readiness impedance of the skilled players. In another work [16], the authors studied the hand impedance measurements comparatively across professional and novice manual welders when they performed tungsten inert gas (TIG) welding interactively with the KUKA lightweight robot arm (LWR). The result showed that hand impedance profiles differed across professional and novice welders, demonstrating the superior capability of the skilled workers in regulating the arm endpoint impedance.

Inspired by the stiffness regulation capability of the human arm, some studies have made an attempt to transfer such an ability to robotic manipulators. In that direction, the first problem to address was the estimation of the human arm endpoint stiffness or joint stiffness, so that either the real-time stiffness profiles or the underlying impedance-regulation principles can be transferred to robotic manipulators. In [17], a practical approach to estimating human wrist stiffness during tooling tasks was proposed, and the joint stiffness information was pre-programmed to allow transferring skills from expert human operators to an impedance-controlled industrial robot. Instead of only modeling the wrist stiffness, modeling human arm endpoint stiffness based on a complete arm skeleton model is more useful in practice. The methods to estimate the human arm endpoint stiffness can be divided into offline and online categories. Stochastic [18] or deterministic [19] perturbation techniques, for instance, are among the offline methods to identify discrete human arm endpoint stiffness in certain arm postures. The alternative offline techniques make use of human upper extremity musculoskeletal models [20], which can generate appropriate muscle activities to repeat a movement recorded from a human demonstration. In such models, human arm joint stiffness is calculated according to the generated muscle activities, and consequently, the endpoint stiffness is formulated through a conservative congruence transformation [21]. The latter technique is suitable for the continuous stiffness profile estimation’ however, the process is very complex, which makes it hardly suitable for applications that require personalized human models.

The online human impedance estimation techniques were first proposed under the concept of teleimpedance control [22], for real-time transferring of human arm stiffness to teleoperated robots. To extend this model to a larger arm workspace, the theory of the common mode stiffness (CMS) and configuration dependent stiffness (CDS) was proposed in [23]. The CMS, which reflected the effect of muscular co-activation in synchronous modulation of the stiffness ellipsoid’s major axes, and the CDS, which reflected the geometric contribution of the arm Jacobian, were used for the estimation of the arm endpoint stiffness projected from joint stiffness in real-time. However, the joint stiffness matrix is a symmetric and non-diagonal matrix, which means for a 7-DOF human arm, there are 28 unknown parameters in this matrix (30 in total, two for the muscular co-activation model). Furthermore, to reduce the open parameters in the model, the muscle Jacobian was introduced in [24]; it leads to a diagonal muscle stiffness matrix. The number of unknown parameters in this matrix depends on the number of selected muscles (11 muscles were selected and 13 unknown parameters in total in [24]). Although this resulted in an less parameterized and accurate model, calculating the muscle Jacobian can be quite complex, hindering its widespread use in practice.

**Figure 1:**
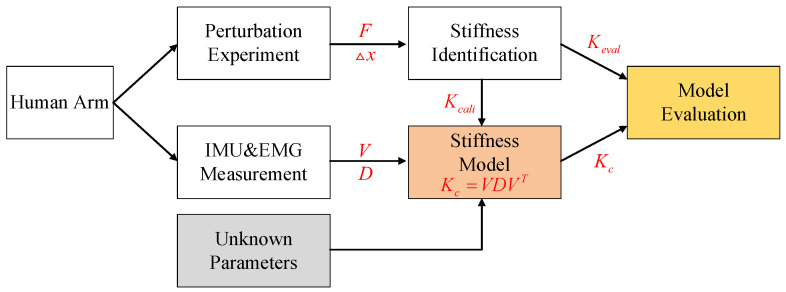
Graphical illustration of the main blocks in this work. F and Δx represent interaction force between human and robot and corresponding displacement respectively. V and D represent the orthonormal matrix consisting of eigenvectors and the diagonal matrix consisting of eigenvalues. $K_{c},K_{cali}$ and $K_{eva}$ represent the estimated human arm endpoint stiffness from the proposed model and the collected human arm endpoint stiffness samples used for calibration and evaluation respectively.

As a continuation of our developments in this direction, in this work, we proposed a new, principally simplified, and intuitive online model of the human arm endpoint stiffness which significantly reduces the total unknown parameters to 4 and without computation of above Jacobians. The new model is based on the large dependency of the shape and orientation of the stiffness ellipsoid on the arm configuration. In fact, previous studies [19,25] indicate that (i) the major principal axis of the arm endpoint stiffness ellipse passes close to the hand-shoulder vector, and (ii) when the arm is extended and the hand moves further from the shoulder, the ellipse becomes more elongated, and conversely, it becomes more isotropic. Inspired by those findings, in this work, we propose a new model in 3D formed by constructing the ellipsoids’ principal axes and their lengths, namely, the eigenvectors and eigenvalues, based on the arm configuration. Compared with the recent model proposed in our previous work [24], we exploit the basic human arm motion data to retrieve arm geometry information, from which the CDS part is constructed. This is in contrast to our previous models that exploited human arm and muscle Jacobians, with the latter being quite complex. This consideration can significantly increase its integration and personalization potential in various application scenarios, such as teleoperation and human kinodynamics tracking for ergonomic assessment. The main blocks of this work are illustrated in Figure 1. Under our experimental setup, through a deterministic perturbation experiment, perturbed force F and displacement Δx were recorded and from which the separate human arm endpoint stiffness samples were collected. The elements V and D in the human arm endpoint stiffness estimation model were constructed based on inertial measurement unit (IMU) and electromyography (EMG) sensors’ measurements and a few unknown parameters. Then, the model’s unknown parameters were calibrated by using the collected stiffness samples for calibration $K_{cali}$. Finally, the accuracy of the proposed model $K_{c}$ was evaluated by using the collected stiffness samples for evaluation $K_{eval}$.

The remainder of this paper is structured as follows: Section 2 elaborates on the theory of the new model. Section 3 starts with the identification of human arm endpoint stiffness, which is then followed by the optimization of the model parameters. In Section 4, the accuracy of the model across several subjects is evaluated. Finally, the discussions and conclusions are presented in Section 5.

## 2. Formulation

**Figure 2:**
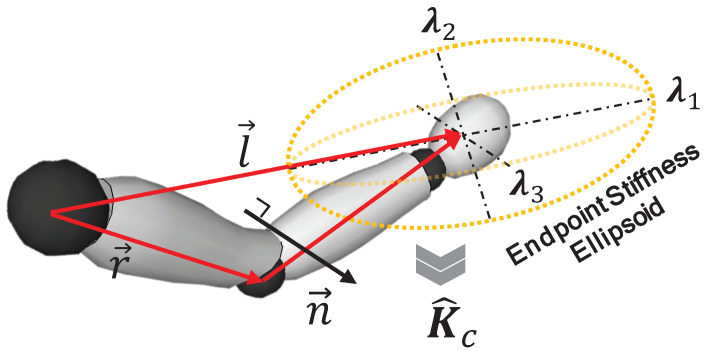
The geometric relationship between the human arm configuration and the principal axes of the endpoint stiffness ellipsoid. $\vec{n}$ is perpendicular to the plane formed by $\vec{l}$ and $\vec{r}$. $\vec{l}$ and $\vec{n}$ are parallel to the major and minor principal axes of the endpoint stiffness ellipsoid, respectively.

As shown in Figure 2, in this work, we use a two-segment human arm skeleton structure in 3D space. The hand–forearm and upper arm segments in this model permit us to form an arm triangle [26] at any non-singular configuration. Relying on the dominant contribution of the arm configuration to the endpoint stiffness geometry, we propose to use the vector from the center of shoulder joint to the position of the hand ($\vec{l}\in R^{3}$), to identify the major principal direction of the human arm endpoint stiffness ellipsoid. The minor principal axis direction ($\vec{n}\in R^{3}$), instead, is defined to be perpendicular to the arm triangle plane

(1) $$\vec{n}=\vec{r}\times \vec{l},$$

where $\vec{r}\in R^{3}$ represents the vector from the center of shoulder to the center of elbow.

The remaining principal axis of the stiffness ellipsoid ($\vec{m}\in R^{3}$), which lies on the arm triangle plane, is calculated based on the orthogonality of the three principal axes

(2) $$\vec{m}=\vec{n}\times \vec{l}=\left(\vec{r}\times \vec{l}\right)\times \vec{l}.$$

Hence, the orthonormal matrix $V\in R^{3\times 3}$ can be constructed as

(3) $$V=\left[\frac{\vec{l}}{\|\vec{l}\|},\frac{\left(\vec{r}\times \vec{l}\right)\times \vec{l}}{\|\left(\vec{r}\times \vec{l}\right)\times \vec{l}\|},\frac{\vec{r}\times \vec{l}}{\|\vec{r}\times \vec{l}\|}\right].$$

From the equation above, when the arm is extended and the hand moves further from the shoulder, the ellipse becomes more elongated, and conversely, it becomes more isotropic. Hence, we assume that the ratio of the length of median principal axis to the major principal axis of the stiffness ellipsoid is inversely proportional to the distance $d_{1}$ from the hand position to the center of shoulder.

(4) $$\frac{\lambda_{2}}{\lambda_{1}}=\frac{\alpha_{1}}{d_{1}},$$

where $\lambda_{1}$ and $\lambda_{2}$ represent the eigenvalues corresponding to the major and median principal axes, respectively. $\alpha_{1}$ is the scale factor and

(5) $$d_{1}=\left\|\vec{l}\right\|.$$

The ratio of the length of the minor principal axis to the major principal axis is assumed to be proportional to the distance $d_{2}$ from the center of the elbow to the major principal axis.

(6) $$\frac{\lambda_{3}}{\lambda_{1}}=\alpha_{2}\cdot d_{2},$$

where $\lambda_{3}$ represents the eigenvalue corresponding to the minor principal axis, $\alpha_{2}$ is the scale factor, and

(7) $$d_{2}=\vec{r}\cdot \frac{\vec{m}}{∥\vec{m}∥}=\vec{r}\cdot \frac{\left(\vec{r}\times \vec{l}\right)\times \vec{l}}{\|\left(\vec{r}\times \vec{l}\right)\times \vec{l}\|}.$$

Previous works indicate that human arm muscles contribute to the endpoint stiffness in a synergistic way. The findings in [19] show the magnitude of the stiffness is coordinately increased but only minor changes occur in shape and orientation when a disturbance occurred to the arm. A force regulation task was introduced in [27] to examine the human arm’s abilities (especially the muscles) regarding voluntary control of static endpoint stiffness with a fixed arm configuration; it turned out that individuals can voluntarily change stiffness orientation but that the magnitude of these changes is small. Therefore, in this work, we built the model based on the assumption of the synergistic contribution of the arm muscle co-contractions to the endpoint ellipsoid’s volume. This contribution is represented by the active component $A_{cc}$.

(8) $$A_{cc}\left(p\right)=c_{1}\cdot p+c_{2},$$

which is in a linear relation with the muscle activation level *p*. $c_{1}$ and $c_{2}$ are two coefficients to be identified.

The diagonal matrix $D\in R^{3\times 3}$ is formed by the length of the principal axes, i.e., the eigenvalues,

(9) $$D=A_{cc}\left(p\right)\cdot D_{s}=A_{cc}\left(p\right)\cdot \left[\begin{array}{ccc} 1 & \; & \\ \; & \alpha_{1}/d_{1} & \\ \; & \; & \alpha_{2}d_{2} \end{array}\right],$$

where $\lambda_{1}$ is set to $A_{cc}$, and $D_{s}\in R^{3\times 3}$ represents the shape of the stiffness ellipsoid. Finally, the estimated endpoint stiffness matrix ${\hat{K}}_{c}\in R^{3\times 3}$ is formulated by

(10) $${\hat{K}}_{c}=VDV^{T}=VA_{cc}\left(p\right)D_{s}V^{T}.$$

In this formulation, only four parameters ($c_{1}$, $c_{2}$, $\alpha_{1}$ and $\alpha_{2}$) must be personalized to match an individual’s physical interaction characteristics. Note that this formulation guarantees the symmetry and positive definiteness of the endpoint stiffness matrix, as long as the eigenvalues are positive. The number of dimensions of the input space of this model is six, which are composed of (1) a one-dimensional vector for the EMG signal, and (2) a three-dimensional vector $\vec{l}$ and three-dimensional vector $\vec{r}$; we use with the elbow-hand vector length constraints which reduce the number of dimensions to five. The output space, which includes the translational Cartesian stiffness components of the endpoint stiffness matrix, has six dimensions as well. The following chapter explains the procedure for the identification and personalization of the model parameters.

## 3. Identification of Model Parameters

The personalization of the arm endpoint stiffness model was achieved by identifying the four previously mentioned model parameters. To achieve this and evaluate the accuracy of the proposed model, first, the endpoint stiffness matrices of the arm in six arm configurations (evenly distributed in the main workspace of human arm) and seven muscle co-contraction levels were estimated through perturbation experiments [19]. (In theory, only four trials are needed to personalize the model parameters. In this work, far more than enough data were collected to identify model parameters and evaluate the accuracy of the proposed model in different arm states.) The identified stiffness matrices were then used for the identification of the model parameters and the validation of the model’s accuracy (using new test data). Six dominant arm muscles, i.e., the anterior and posterior portions of the deltoid, the biceps brachii, the triceps brachii, the brachioradialis and the flexor carpi ulnaris, were grouped in three antagonistic configurations and the subjects were asked to voluntarily keep each muscle group’s co-contraction levels in three states: relaxed, 10% and 20% of the maximum voluntary contraction (MVC). This resulted in a total number of 84 trials per subject.

Four healthy subjects (3 males and 1 female, 20 to 37 years of age (subjects had no neurological or orthopedic impairment of the upper limbs and gave informed consent to all procedures and were free to withdraw from the study at any time)), participated in the model validation experiments.

### 3.1. Human Arm Endpoint Stiffness Identification

#### 3.1.1. Experiment Setup

**Figure 3:**
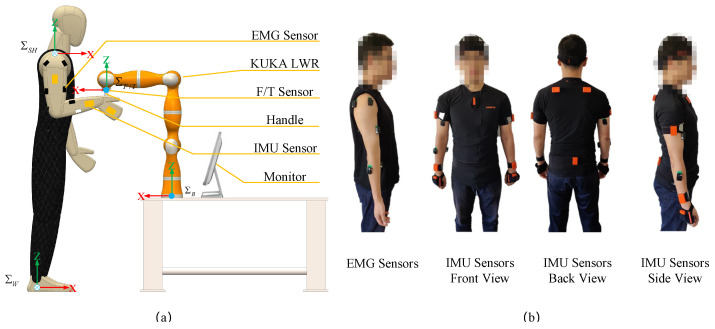
(**a**) The experiment setup for human arm endpoint stiffness identification. A fixed-base KUKA light-weight robot, equipped with a six-axis force/torque sensor at the end-effector, was used to apply perturbations to the human hand (note that arm endpoint movements were measured by high resolution encoders of the KUKA robot, which was active in position control mode). (**b**) EMG and IMU sensor setup. Sux EMG sensors were attached to the corresponding muscles’ places on the right arm and 11 IMU sensors were placed for upper body movement recording according to MVN Biomech suit protocol.

The experimental setup is illustrated in Figure 3a, and the local reference frames of the model are defined in here as well: the world frame $\Sigma_{W}$, the shoulder frame $\Sigma_{SH}$, the KUKA base frame $\Sigma_{B}$ and the F/T frame $\Sigma_{F/T}$. During the experiment, the subjects stood in front of a KUKA LWR robot with their right hand grasping an aluminum handle, mounted to a force/torque sensor—which was used to measure the perturbed force and had a sampling rate of up to 3 kHz (ATI, mini45, EtherCAT interface)—and attached to the end effector of the robot. The robot was kept rigidly fixed through an anti-vibration table. A wearable MVN Biomech suit (Xsens Technoloies BV), which is based on 11 inertial measurement unit (IMU) sensors (only for the upper body, 3 axes for measurement and update frequency of 60~80 Hz), was used to measure the motion of the two arms of the subject. It was employed to calculate the vectors $\vec{l}$ and $\vec{r}$. We assume the human model to correspond to $\Sigma_{W}$, which was fixed at the heel of subject’s right foot. $\Sigma_{SH}$ was used to record the orientation of the shoulder with respected to $\Sigma_{W}$, and the human’s right arm motion was represented in $\Sigma_{SH}$.

Six electromyography (EMG) sensors whose sampling rate was up to 1 kHz (Delsys, Trigno Wireless Biofeedback System) were attached to the right arm’s selected muscles to record their activities. They were divided into three groups, i.e., the shoulder muscle group (with the anterior and posterior portions of the deltoid), the upper arm muscle group (with the biceps brachii and the triceps brachii) and the forearm muscle group (with the brachioradialis and the flexor carpi ulnaris). The EMG and IMU sensor setup is displayed in Figure 3b. During the experiments, the left half of the back of the subjects were fixed against the wall, to stabilize the trunk and shoulder joints of the right arm. A monitor provided the real-time visual feedback about EMG information to the subjects during the experiments.

**Figure 4:**
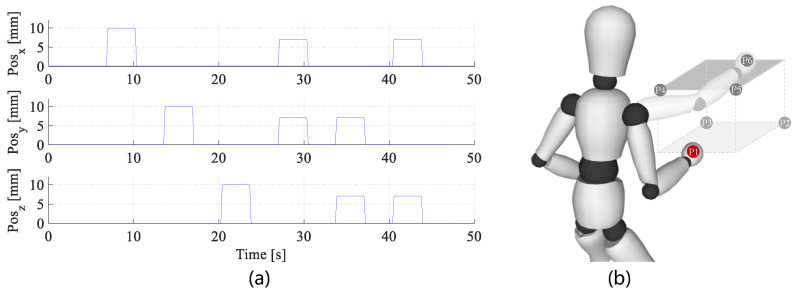
Experimental protocols. (**a**) A typical perturbation trajectory profile with the desired displacement of 10 mm in the target directions (*x*, *y*, *z*, x−y, y−z, x−z). (**b**) Predefined equilibrium positions for the perturbation experiments. These positions were selected in two planes, from P1 to P6. In each plane, there were three positions that formed a triangle.

The KUKA robot was controlled in the position mode by an external PC through the fast research interface (FRI), in which a set of perturbation trajectories (36 in total, see Figure 4a for a typical perturbation trial) was stored. Six types of perturbation movements in different directions were included in each trajectory, with a randomized order of directions (*x*, *y*, *z*, x−y, y−z, x−z) to avoid voluntary responses before each perturbation. The profile was generated under a snap (second time derivative of acceleration) control law to make sure it was smooth enough. All perturbation trials had similar durations of about 350 ms, and the maximum displacement was set to 10 mm.

The EMG data, the F/T data and the human motion data were collected and streamed to the external PC through User Datagram Protocol (UDP). The end effector position of the KUKA LWR was also stored in the external PC, where all the data were synchronized and saved.

#### 3.1.2. Experiment Protocols

After wearing MVN Biomech suit and the EMG sensors, the subjects were required to contract each muscle separately to retrieve the MVC values. In the experiments, the processed (fully rectified and filtered) EMG signals were divided by the corresponding MVC values and the resulting percentages were shown visually to the subjects. Before starting the experiment for each trial, the MVN Biomech suit was calibrated with N-pose (i.e., two arms straight down, palms facing to their body, feet parallel to each other and apart) to initialize the skeleton mode.

To take into account the effect of muscular co-contractions on the arm endpoint stiffness, as noted before, three muscle groups were selected. In each arm configuration, for the first trial, the subjects were asked to keep the muscles as relaxed as possible. Instead, from the second to the seventh trial, they were asked to co-contract one muscle group at a time, and keep it at a specific level (i.e., shoulder 10% MVC, shoulder 20% MVC, upper arm 10% MVC, upper arm 20% MVC, forearm 10% MVC, forearm 20% MVC) by following the real-time feedback guidance, while relaxing the other two groups as much as possible.

To make the experiment friendly to the subjects and guarantee that the samples covered most workspace of the human arm, we made a compromise for the number of positions. Here six equilibrium positions (subjects were asked to keep the same arm posture among different muscle activation states when at one position.) were predefined in the common workspace of the robot and the subject’s right arm, as shown in Figure 4b. All the positions avoided singular positions for human and the robot.

Each trial was repeated at least two times—the first one for the model training and the other one for the evaluation. For each subject, at least 84 (=6×(3×2+1)×2) trials were recorded (note that a very small portion of this data were necessary for the personalization of the model, which will be described later; the rest of this data were meant for the model validation.). Trials from which the estimated stiffness matrix did not satisfy positive definite and symmetric conditions were discarded and repeated. The whole experimental procedure was conducted in accordance with the Declaration of Helsinki and the protocol was approved by the ethics committee Azienda Sanitaria Locale Genovese (ASL) N.3 (Protocol IIT_HRII_001).

#### 3.1.3. Data Processing

Raw data collection of IMU and EMG sensors was through wireless network and it was filtered online by a (finite impulse response) FIR filter and an internal filter respectively. The filtered data was used as the input of the proposed model in the following.

**Figure 5:**
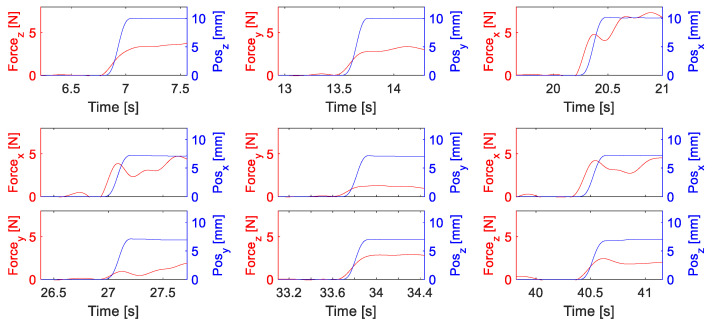
Typical results of a perturbation experiment to identify the arm endpoint stiffness matrix. The displacement profile (blue) and the measured reaction forces (red) are illustrated for one selected trial. Subplots in the first row from left to right represent perturbation in *z*, *y* and *x* directions, respectively. Subplots in the columns of the remaining two rows from left to right represent perturbations in x−y, y−z and x−z directions, respectively.

The estimation of the human arm endpoint stiffness, which was used for model calibration and evaluation, was achieved by applying a displacement profile and the measurement of the restoring forces. Figure 5 illustrates typical examples of the displacement and force data. The arm endpoint displacements were recorded through the high resolution encoders of the KUKA LWR robot and a forward kinematics calculation. The response forces were measured by an external 6-axis F/T sensor and the raw data were streamed through an Ethernet cable by using the EtherCAT interface. A Butterworth filter was used to remove the noise of F/T data offline. The offset force for each perturbation was removed by using an average of the data during 0.5 s before the perturbation started.

The three diagonal terms of the translational Cartesian stiffness matrix were calculated by the *x*, *y* and *z* perturbation profiles. Next, the off-diagonal elements were calculated by using the data from x−y, x−z and y−z directional displacements.

The human arm endpoint stiffness matrices estimated from the displacements and forces are represented in $\Sigma_{B}$. Since the human arm motion data were represented in $\Sigma_{SH}$, we transformed the translational stiffness matrices from $\Sigma_{B}$ to $\Sigma_{SH}$ as follows

(11) $$K_{SH}=\left(R_{W}^{SH}R_{B}^{W}\right)K_{B}{\left(R_{W}^{SH}R_{B}^{W}\right)}^{T},$$

where $K_{SH}\in R^{3\times 3}$ and $K_{B}\in R^{3\times 3}$ represent human arm endpoint stiffness in $\Sigma_{SH}$ and $\Sigma_{B}$ respectively; $R_{B}^{W}\in R^{3\times 3}$ and $R_{W}^{SH}\in R^{3\times 3}$ represent the rotation matrices from $\Sigma_{B}$ to $\Sigma_{W}$ and from $\Sigma_{W}$ to $\Sigma_{SH}$, respectively.

**Figure 6:**
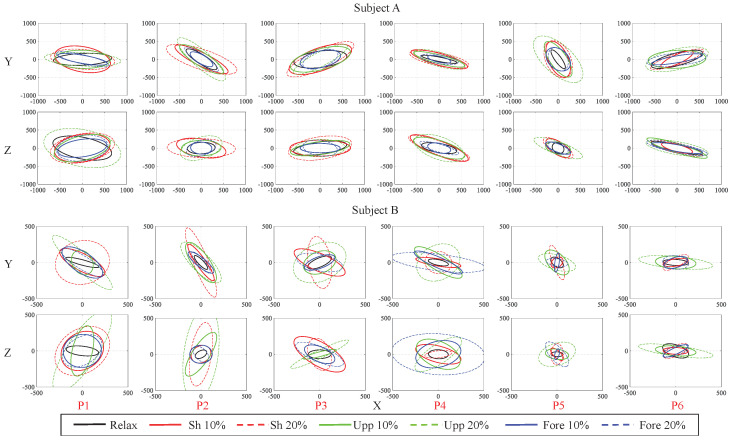
The estimated Cartesian stiffness ellipsoids of subjects **A** and **B** (typical results across subjects) in different arm configurations and muscle co-contraction states. P# represents an individual arm pose, and the different colors in the subplot represent different muscle co-contraction states, as shown in the bottom legend.

Figure 6 shows typical results across subjects of the estimated human arm endpoint stiffness $K_{SH}$ projected on X–Y and X–Z planes for subjects A and B in different arm configurations (from P1 to P6) and muscle co-contraction states (i.e., all relaxed and shoulder 10% MVC, shoulder 20% MVC, upper arm 10% MVC, upper arm 20% MVC, forearm 10% MVC and forearm 20% MVC, while relaxing the other two groups as much as possible).

### 3.2. Identification of Model Parameters

As already mentioned in Section 2, there are four parameters ($c_{1}$, $c_{2}$, $\alpha_{1}$ and $\alpha_{2}$) in the proposed model that must be identified. $c_{1}$ and $c_{2}$ are the two coefficients in the linear model of the active component $A_{cc}$. $\alpha_{1}$ and $\alpha_{2}$ are the body dimension-related ($d_{1}$ and $d_{2}$ respectively) coefficients.

The identification of these parameters was achieved by minimizing the objective function with the training data set (N=42):

(12) $$\min\sum_{i=1}^{N}{∥\log\left(V_{i}A_{cci}D_{si}V_{i}^{T}\right)-\log\left(K_{SHi}\right)∥}_{F}.$$

In this process, instead of the Frobenius norm, the log-Frobenius norm was adopted due to the SPD property of the stiffness matrix. The space for the stiffness matrix is nonlinear, which in fact belongs to the SPD Riemannian manifold $S_{++}^{D}$ [28]. The minimum length curves between two points on a Riemannian manifold are called geodesics, which is similar to straight lines in Euclidean space. Both affine-invariant and log-frobenius distance are geodesics for $S_{++}^{D}$ [29]. To guarantee positive definiteness of the modeling stiffness matrix and the correspondence of eigenvectors and eigenvalues, we considered lower and upper bounds for $\alpha_{1}$ and $\alpha_{2}$ in the optimization.

To prepare the required data for the optimization, the measured human arm endpoint stiffness $K_{SH}$ was first computed. Through the motion data of the centers of shoulder and elbow and the hand position, the vectors $\vec{l}$ and $\vec{r}$ were computed and then the orthonormal matrix V was constructed by (3). The distances $d_{1}$ and $d_{2}$ were calculated based on (5) and (7) respectively, to construct the diagonal matrix $D_{s}$.

**Figure 7:**
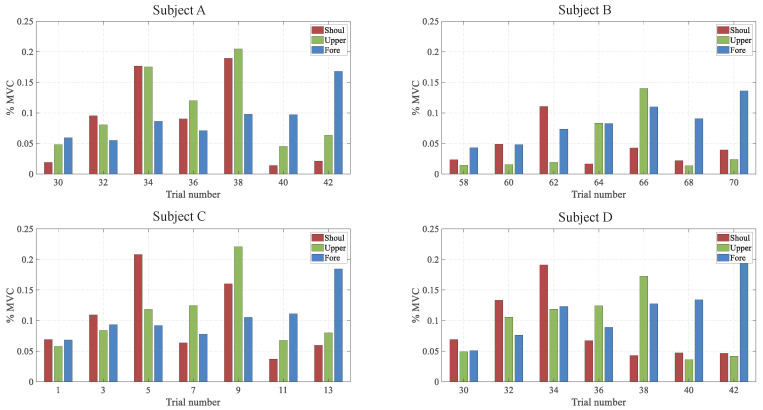
Percentage of MVC value of each muscle group for every subject at one selected position. The colors red, green and blue respectively represent the activation level of the shoulder muscle group, upper arm muscle group and forearm muscle group.

The input of the active component $A_{cc}$, was computed by filtering the selected EMG signals, from the data segment which corresponded to the same period of perturbation experiments. In Figure 7, each subplot illustrates the %MVC values of each muscle group for different trials in one selected arm configuration. It is evident that the activity of the muscle groups mainly followed the indicated experimental protocols that ranged from a relaxed state to 10% and 20% MVC. Three muscle groups, i.e., the forearm, the upper arm and the shoulder, were the candidates to model $A_{cc}$. Considering that the forearm and the shoulder muscle groups are distributed far from each other in the arm, their activity may be less correlated, which means that the upper arm muscle group could be a good choice for tracking the co-activation pattern of the arm muscles. In fact, as observable in Figure 7, the activity of the upper arm muscle group demonstrates a closer relationship to the other two muscle groups. Thus, we chose the activity of the upper arm muscle group as the EMG signal input for the model. Finally, we built the terms in Equation (12) with four unknown parameters from the collected data. The optimisation was performed by the fmincon function (MATLAB, The MathWorks Inc.).

## 4. Results

The identified model parameters for the four subjects are listed in Table 1. The average (AVG), standard deviation (SD) and coefficient of variation (CV) of the parameters were calculated across subjects. From the CV values of different parameters, it can been seen $c_{1}$ and $c_{2}$ exhibit large difference while $\alpha_{1}$ and $\alpha_{2}$ are similar within subjects. The reason behind is that $\alpha_{1}$ and $\alpha_{2}$ are closely related to the arm skeleton dimensions and which do not have much difference within the subjects in our experiment. $c_{1}$ and $c_{2}$ are closely related to the strength of the muscles, which may have large difference across the subjects.

To evaluate the accuracy of the proposed model, the following function (13), where $I_{3}$ represents the 3 × 3 identity matrix, was calculated:

(13) $$E_{K}=\frac{1}{N}\sum_{i=1}^{N}\frac{∥\log\left(V_{i}A_{cci}D_{si}V_{i}^{T}\right)-\log\left(K_{SHi}\right){∥}_{F}}{∥\log\left(K_{SHi}\right)-\log\left(I_{3}\right){∥}_{F}}.$$

By utilising the test data set (N=42) from different subjects, the model error was within 13%∼31%, and the average error was around 20%.

**Figure 8:**
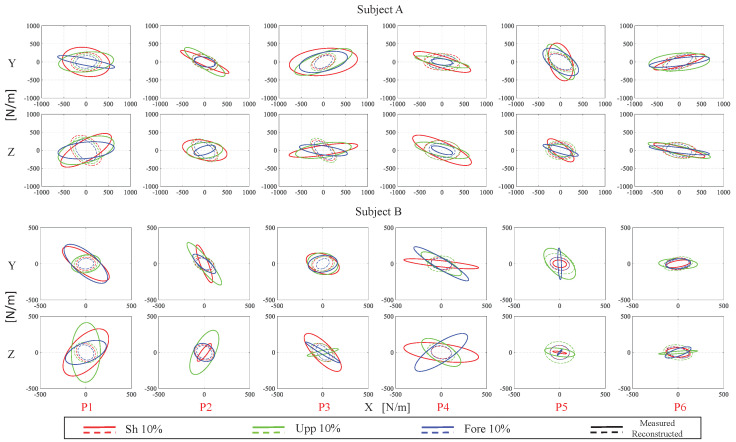
A comparison between the measured endpoint stiffness (solid line), which was calculated from the displacement profile and the reaction forces in the perturbation experiment, and the predicted one (dashed line), which was estimated by our model for subjects **A** and **B** (typical results across subjects) at different arm configurations and muscle activation states. P# represents different positions; the different colors in the subplot represent different muscle activation states, as shown in the bottom legend.

Figure 8 demonstrates the typical results across subjects of the measured (solid line) and the predicted (dashed line) endpoint stiffness ellipses on X–Y and X–Z planes for the subjects A and B in 10% MVC of the shoulder, upper arm and forearm muscles. As illustrated, the principal axis directions of the reconstructed (from model) ellipses largely match the estimated (from experiments) ones.

To provide a quantified comparison, the angle between the major principal axes of the measured and the predicted endpoint stiffness ellipsoids is used to evaluate the directional mismatch between the two. In addition, the relative error of the volume of the measured and the predicted endpoint stiffness ellipsoids is used to evaluate the size difference. To keep in line with the data depicted in Figure 8, the above two indexes were separately analyzed on X–Y and X–Z planes. For the principal axes difference, the average angle errors on X–Y plane across test data set for subjects A, B, C and D were 0.2870±0.2058 rad (the data are reported as: mean ± standard deviation), 0.4540±0.2973 rad, 0.3155±0.1926 rad, and 0.6389±0.3613 rad, respectively, resulting in a total average angle error of 0.4238±0.1609 rad across all subjects. On X–Z plane, the average angle errors for subjects A, B, C and D were 0.6324±0.5074 rad, 0.6347±0.4858 rad, 0.7183±0.4834 rad, and 0.6736±0.4192 rad, respectively, resulting in a total average angle error of 0.6648±0.0404 rad across all subjects. Compared with the error on X–Y plane, the error on X–Z plane is a bit higher due to more uncertainty existing in Z direction caused by the neglected gravitational effect of the human arm. This confirms and gives credibility to our geometric approach in constructing the eigenvectors of the stiffness model.

On the other hand, for the size difference, the average relative error of ellipses area δ is defined by (14) for each subject. It resulted in δ equal to 0.4093±0.2517,0.5200±0.1997,0.4180±0.2477 and 0.5576±0.2955 for subjects A, B, C and D on X–Y plane, respectively, and therefore a total average error is 0.4763±0.0740 across all subjects. On X–Z plane, the average relative error δ was equal to 0.3003±0.1944,0.5916±0.2952,0.5081±0.2369 and 0.6410±0.3143 for subjects A, B, C and D, respectively; the total average error therefore, was 0.5102±0.1503 across all subjects. Regarding the shape/volume, the reconstructed ellipses were more circular than the measured ones and because of this, the volumes cannot match each other very well after the optimization. Most of the reconstructed ellipses were smaller than the measured ones mainly in *x* direction. One potential limitation is that the shape/volume mismatch may cause the reducing of modeling accuracy because of the movement of subject’s trunk was rigidly constrained only in *x* direction, where is in the experimental setup. In fact, in [30], the effects of unconstrained trunk and shoulder movements on arm endpoint stiffness were examined, it showed that the compliance associated with unrestrained trunk and shoulder decreased the static endpoint stiffness of the arm. Thus, the true human arm endpoint stiffness values in *y* and *z* directions should be larger than the measured ones. It means that the true stiffness ellipses could be more circular and the shape/volume mismatch problem could be relieved.

(14) $$\delta =\frac{1}{N}\sum_{i=1}^{N}\left|\frac{S_{pi}-S_{mi}}{S_{mi}}\right|,$$

where δ is the average relative error of the measured and the predicted ellipses area across the test data set for each subject, $S_{pi}$ and $S_{mi}$ are respectively the *i*-th predicted and measured ellipses area on X–Y or X–Z plane.

## 5. Discussion and Conclusions

In this paper, we proposed a new human arm endpoint stiffness model, in which the stiffness matrix $K_{c}$ was decomposed as $K_{c}=VDV^{T}$ through eigendecomposition. The unitary basis V and the singular values of D were extracted directly from a reduced human arm skeleton structure in 3D and a muscular co-contraction index, respectively.

The proposed model offers several advantages. Firstly, the calibration and personalization of the model to an individual can be achieved through the identification of only four parameters. This implies that a small-scale experiment’s data would be sufficient to calibrate this model to match an individual’s physical characteristics. As it was shown in the last column of Table 1, the average values of $\alpha_{1}$ and $\alpha_{2}$ were used to re-evaluate the model accuracy. It turns out that minor error is increased compared with the personalized $\alpha_{1}$ and $\alpha_{2}$ which means in some specific applications where only CDS is considered, the average value could be used even without parameter identification process for a new individual who shares a similar arm skeleton dimensions with the subjects in our experiment. Secondly, the SPD properties of the calculated arm endpoint stiffness are guaranteed, which is due to the underlying eigendecomposition. Thirdly, and relying on the above two advantages, the log-frobenius, which is the geodesic length between two elements in $S_{++}^{D}$, can be used in the optimization procedure to speed up the convergence rate. Lastly, due to the above advantages, the implementation of this model requires less programming work compared with previous models. The main difference appears in the CDS part. In [23,24], the input for CDS is the joint angles which means an inverse kinematics process has to be preprogrammed based on the raw data from the IMU, and a 3 by 7 matrix of arm Jacobian has to be constructed in [23] and besides that, a 7 by 11 matrix of muscle Jacobian is also needed in [24] which requires the forward kinematics of the length of relative muscles respect to the joint angles and it is hard to develop from scratch for most researchers. However, in the proposed model, the input for CDS is the raw data from IMU without any inverse kinematic process. Meanwhile, the construction the eigenvectors and eigenvalues is much easier compared with the Jacobians, especially the muscle Jacobian. This advantage makes our model more suitable for human–robot interaction and collaboration, and teleoperation (teleimpedance) applications. In robot learning from demonstration scenarios, by employing this model, one can enrich the demonstrated data by adding human stiffness profile to the kinodynamic data that can be measured by external sensory systems.

The price paid for the above substantial advantages is the average model error of around 20% evaluated by (13) which mainly catches the nonlinear structure of SPD stiffness matrix, although the average size difference evaluated by (14) is around 50% which is closely related to the linear part of the stiffness matrix. This error is mainly introduced by neglecting the effect of external forces [14] and probably by our assumption on muscle synergies [31]. Therefore, the error is imposed by the trade-off between simplicity and accuracy of the model. Noteworthy is that the influence of the external forces on human arm stiffness is also ignored in [24].

The model error is still acceptable for most applications mentioned before. In particular, for what concerns the stiffness ellipsoid’s geometry, according to the quantitative comparison in principal axes difference between the measured and predicted stiffness in last section, the constructed principal axes coincide with the experimentally measured ones with a limited error. In most applications, the stiffness geometry plays a dominant role compared to its volume. In fact, some tasks do not require specific stiffness values to be executed, but stiffness ranges along principal axes. An example is the assembly tasks (e.g., peg-in-hole), where the stiffness profile along the insertion must be higher than the others to comply with the environmental constraints. For this kind of application, the measurement error in the proposed model may not result in task failure. However, there are other applications that are more sensitive to the variation of stiffness values, which means that the task may fail because of the existence of a similar error. In this case, only relying on the task-related stiffness information recorded from human arm may be not sufficient. Nonetheless, by combining adaptive control approaches or reinforcement learning algorithms, a fine modulation of the modeled stiffness profile is expected to compensate for such an error.

## Figures and Tables

**Table 1:** Model parameters’ identification results.

Subjects	*c*_1_	*c*_2_	*α*_1_	*α*_2_	Error	Error (avg *α*_1_ and *α*_2_)
A	1539.478	238.313	0.245	2.712	13.25%	13.34%
B	1015.907	99.567	0.296	3.231	21.67%	21.69%
C	3942.261	152.281	0.252	1.892	17.00%	17.74%
D	1635.365	72.262	0.230	3.428	30.83%	31.12%
AVG	2033.325	140.606	0.255	2.815	20.69%	20.97%
SD	1301.465	73.116	0.028	0.6859	-	-
CV	64.01%	52.00%	11.09%	24.36%	-	-
